# Medial Expression of TNF-α and TNF Receptors Precedes the Development of Atherosclerotic Lesions in Apolipoprotein E/LDL Receptor Double Knockout Mice

**Published:** 2007-06

**Authors:** Audrey Niemann-Jönsson, Ingrid Söderberg, Marie W Lindholm, Stefan Jovinge, Jan Nilsson, Gunilla Nordin Fredrikson

**Affiliations:** 1 *Department of Clinical Sciences, Malmö University Hospital, Lund University;*; 2 *Department of Cardiology, Lund University Hospital and Lund Stem Cell Center, Lund University;*; 3 *Department of Biomedical Laboratory Sciences, Malmö University, Sweden*

**Keywords:** atherosclerosis, lipoproteins, TNF-α, TNF receptors

## Abstract

TNF-α is present in atherosclerotic lesions, activates endothelial adhesion molecule expression, stimulates the release of proinflammatory cytokines and matrix metalloproteinases and promotes smooth muscle cell proliferation and migration. Taken together these observations suggest that TNF-α may be functionally involved in early atherosclerosis development. To further evaluate this hypothesis we compared vascular TNF-α and TNF receptor expression in atherosclerosis-susceptible apoE^-/-^/LDL receptor^-/-^ mice and control C57BL/6 mice. The aortas of 8 week old apoE^-/-^/LDLreceptor^-/-^ mice displayed immunoreactivity for TNF-α as well as TNF p55 and p75 receptors (2.1 ± 1.6%, 5.6 ± 1.5% and 3.6 ± 1.3% of total media area, respectively), but did not have any detectable lesions. A marginal increase in TNF-α and TNF receptor immunoreactivity was observed at 12 weeks and atherosclerotic plaques were detected in 1 out of 5 animals. At 16 weeks TNF-α expression in the media was increased more than four-fold as compared with 8 week old mice, and atherosclerosis was widespread. TNF-α immunoreactivity was also observed in all plaques. In addition, at the same age a tendency towards increased TNF-α mRNA levels was detected in the double knockout mice compared to age-matched controls. A further increase in TNF-α and TNF receptor immunoreactivity as well as plaque size was observed at 20 weeks. With only a few exceptions, no TNF-α or TNF receptor immunoreactivity was detected in C57BL/6 control mice. These findings demonstrate that medial TNF-α and TNF receptor expression precedes lesion formation in apoE^-/-^/LDL receptor^-/-^ mice.

## INTRODUCTION

TNF-α is a multifunctional cytokine that plays an important role in immune defense (1, 2). It has also been implicated in pathogenesis of several inflammatory diseases (3, 4). TNF-α shares modes of actions with several other pro-inflammatory cytokines but experiences from treating rheumatoid arthritis patients with TNF-α inhibitors suggests that this cytokine is of unique and critical importance in chronic inflammatory disorders (4, 5).

Several lines of evidence indicate that TNF-α, as well as other inflammatory cytokines, may also be involved in the development of atherosclerosis. TNF-α expressed in skeletal muscle and adipose tissue inhibits phosphorylation of the insulin receptor (6-9). By inducing insulin resistance TNF-α may cause metabolic disturbances associated with increased cardiovascular risk, such as low HDL cholesterol levels, hypertriglyceridemia and impaired fibrinolysis (10). Increased plasma levels of TNF-α have been observed in subjects with insulin resistance as well as in patients with early onset of coronary heart disease. Moreover, do the levels correlate with disturbances in the triglyceride as well as glucose metabolism (10, 11).

TNF-α may also have a direct influence on development and progression of atherosclerotic lesions. It is expressed during intimal thickening in humans and in atherosclerotic plaques, and to a lesser extent also in the underlying media (12, 13). Experimental studies have identified several mechanisms by which TNF-α may promote atherogenesis including endothelial adhesion molecule expression (14), activation of macrophages (1), stimulation of smooth muscle cell (SMC) proliferation and migration (15) as well as induction of apoptosis (16). In balloon-injury models of neointima formation, TNF-α is expressed by proliferating SMC (17, 18). Furthermore is the development of transplant atherosclerosis in rabbits inhibited by infusion of TNF-α blocking antibodies (19). In addition, apolipoprotein E (apo E) deficient or apo E/TNF-α double knock-out mice treated with recombinant soluble TNF receptor I releasing pellets demonstrate a significant reduction of lesion size. Almost all of this effect could be retained by only having TNF-α depleted inflammatory cells (20). However, mice lacking either of the TNF p55 or p75 receptors show no evidence of reduced atherosclerosis (21) indicating that the role of TNF-α in atherosclerosis development rewards further investigation. On the other hand, the latter study was performed in a non-atherosclerosis prone animal model (21).

The double knockout mouse model used in the present study lacks both apo E and the low density lipoprotein receptor (apoE^-/-^/LDL receptor^-/-^). This model has been found to have total cholesterol levels similar to the ones of apo E deficient mice, whereas LDL receptor deficient mice have much lower levels (22). In addition, it develops atherosclerotic plaques sufficiently quickly in the absence of cholesterol-enhanced diet in contrast to both the single knockout mouse models. Furthermore, it has a lipoprotein profile more human than that of the apo E single knockout mouse (22).

So far, mechanism for induction of vascular TNF-α expression has not been fully elucidated. In an established athero-lesion, interferon-γ producing T cells may activate TNF-α expression in surrounding cells. Low levels of oxidized LDL stimulate release of TNF-α from macrophages (23). We have previously demonstrated that a transient accumulation of LDL in rat aorta is associated with induction of TNF-α expression by medial SMC (24). The present study was designed to investigate if vascular TNF-α expression precedes plaque formation in a mouse model of lipid-induced atherosclerosis.

## MATERIAL AND METHODS

Male apolipoprotein E and LDL receptor gene double knockout mice (apoE^-/-^/LDL receptor^-/-^, n=31) on a C57BL/6 background and C57BL/6 (n=32), were obtained from Bomice (B&M, Aarhus, Denmark). Animals were fed standard R3 chow (AnalyCen, Lidköping, Sweden).

All mice were killed at mid-day by intraperitoneal injection of 150-300 µl hypnorm: dormicum (1:2 parts) and exsanguinated by cardiac puncture on weeks 8, 12, 16 and 20. The blood was immediately placed on ice and allowed to coagulate overnight at 4°C. Hearts and aortas were surgically removed. Hearts, including the aortic arch and the upper part of descending aorta were washed in 0.9% saline containing 0.02 mM butylated hydroxytoluene (BHT) and fixed in 4% formaldehyde for 12h. For immunohistochemistry, the aortic arch, the thoracic and abdominal regions of the aorta above the renal artery were cut into equal pieces and mounted together in one paraffin block. The abdominal aorta below the renal artery and the common iliac arteries were immediately placed in liquid nitrogen and used for mRNA extraction.

### Immunohistochemistry

Tissue sections were deparaffinized with xylene and dehydrated with graded ethanol. The membranes were permeabilized in 0.2% Triton X-100. Endogenous peroxidase activity was quenched by incubating the sections in 0.3% H_2_O_2_ in 80% methanol for 30 min at room temperature. After washing, sections were blocked with 2-5% rabbit serum in PBS for 30 min. Primary antibodies against TNF-α or TNF receptor p55 and 75 (Santa Cruz Biotechnology) were diluted in PBS (1:100 dilution) and incubated for 16 h at 4°C in a humidified chamber. Sections were washed and incubated with biotinylated secondary rabbit anti-goat antibody (Vector Laboratories, Burlingame, California) diluted in PBS (1:200 dilution) for 30 min and then washed. The sections were incubated for 30 min with a peroxidase labeled avidin-biotin complex (Vector) and washed again. Sections were developed using DAB detection kit (Vector) and counterstained in hematoxylin. Negative controls included substitution of the primary antibody with either PBS or irrelevant antibody.

The TNF immunoreactivity was evaluated by image analysis and expressed as percent positive immunostaining of total media or plaque area. A mean medial staining for each animal was calculated based on analyses of 2 sections from the aortic arch, 2 from the descending thoracic aorta and 2 from the abdominal aorta. A mean plaque area for each animal was calculated based on analyses of 6 sections from the aortic arch, 6 from the descending thoracic aorta and 6 from the abdominal aorta.

### Lipid and lipoprotein measurements

Cholesterol and triglyceride concentrations in serum were assayed by colorimetric enzymatic techniques (Sigma, St Louis, Missouri). After an overnight incubation at 4°C the blood samples were spun at 2000 rpm for 10 minutes after which the plasma was removed and stored at -70°C. For analysis, each sample was thawed and diluted (1:10) with PBS and assayed according to instruction using Sigma diagnostics kit.

### RNA isolation and cDNA synthesis

Total RNA was isolated from the aorta from each mouse using the protocol for FastPrep™ system (BIO 101, Carlsbad, California) with small modifications. A speed rating of 6 for 45 seconds to lyse the aortas was used. The synthesis of cDNA was performed by mixing 1 µg of total RNA with random primers (GibcoBRL, Life Technologies, Gaithersburg, Maryland), DTT (GibcoBRL), dNTP (Boeringer Mannheim, Mannheim, Germany), RNaseINH (Promega, Madison, WI, USA) and Moloney Murine Leukemia Virus Reverse Transcriptase (MMLV-RT) (GibcoBRL). Tubes were incubated for 10 min at 30°C, 50 min at 42°C and 2 min at 94°C.

### Real Time Polymerase Chain Reaction

TaqMan Universal PCR Master Mix (Applied Biosystems, Foster City, California) was used in an ABI PRISM 7700 Sequence Detection System (SDS-Perkin Elmer, Applied Biosystems) for amplification of cDNA according to the manufacturer’s protocol. Samples without MMLV-RT in the cDNA synthesis and wells with no template were used as controls. All samples were run in triplicates. Amplification of TNF-α and 18S rRNA was carried out by using cDNA and TaqMan Pre-developed Assay Reagents (PDARs, Applied Biosystems) and 2x TaqMan Universal PCR Master Mix. Amplification of TNF p55 and p75 receptor was carried out by using appropriate concentrations of specific primer pairs and probes and 2x TaqMan Universal PCR Master Mix. The endogenous control 18S rRNA was used for normalization of data. Calculations were performed using standard curves established by serial dilutions of cDNA synthesized from RNA extracted from aortas of seven months old apoE^-/-^ mice (B&M).

### Statistical analysis

Values are given as mean ± SD. Differences between groups were calculated using ANOVA. *P*≤0.05 was considered statistical significant.

## RESULTS

One of the aims of the present study was to compare vascular TNF-α and TNF receptor expression in a mouse strain that spontaneously develops atherosclerosis (apoE^-/-^/LDL receptor^-/-^ mice) with control mice that does not develop atherosclerosis unless challenged with a high-fat diet (C57BL/6). Plasma triacylglycerol and cholesterol levels in apoE^-/-^/LDL receptor^-/-^ and C57BL/6 mice are presented in Table 1. Triacylglycerol levels were 2-3-fold higher and cholesterol levels 4-6-fold higher in the apoE^-/-^/LDLR^-/-^ mice than in the control mice. Both triacylglycerol and cholesterol levels increased with age in the double knockout mice.

**Table 1:** Lipid levels in C57BL/6 and apoE^-/-^/LDLR^-/-^ mice

	Plasma triacylglycerol (mg/mL)		Plasma cholesterol (mg/mL)	
Age (weeks)	Control	DKO	Control	DKO

8	2.09 ± 0.64	3.53 ± 0.59**	1.05 ± 0.14	4.57 ± 0.58***
12	1.83 ± 0.32	5.32 ± 1.92**	1.20 ± 0.10	7.78 ± 0.35***
16	2.02 ± 0.13	6.65 ± 3.83**	1.30 ± 0.10	8.80 ± 1.75***
20	1.93 ± 0.47	6.05 ± 2.70***	1.18 ± 0.14	8.74 ± 2.98***

Control; C57BL/6 mice, DKO; apoE^-/-^/LDLR^-/-^ mice,

** *P*≤0.01 and

*** *P*≤0.001 versus control mice (n=5-6 per group for 8 and 12 weeks old mice and n=10-11 per group for 16 and 20 weeks old mice).

Vascular TNF p55 and p75 receptor expression was present in all apoE^-/-^/LDL receptor^-/-^ mice already at 8 weeks of age. Expression of TNF-α in the aorta was present in 4 out of 6 double knockout mice at this age. In contrast, no staining for TNF-α or TNF receptors was observed at this age in C57BL/6 mice (Table 2). With only a few exceptions C57BL/6 mice remained negative for TNF-α and TNF receptor immunoreactivity throughout the study. Analysis of the apoE^-/-^/LDL receptor^-/-^ mice demonstrated positive staining for TNF-α in 27 out of 31 animals and 30 and 29 for the TNF p55 and p75 receptors, respectively (Table 2). The numbers of mice given here are the sum of all time points (8, 12, 16 and 20 weeks of age).

**Table 2:** Expression of TNF-α and TNF receptors in C57BL/6 and apoE^-/-^/LDLR^-/-^ mice

	TNF-α		TNF p55 receptor		TNF p75 receptor	
Age (weeks)	Control	DKO	Control	DKO	Control	DKO

8	0/6	4/6	0/6	6/6	0/6	6/6
12	3/5	4/5	0/5	4/5	0/5	5/5
16	1/10	10/10	1/10	10/10	3/10	10/10
20	0/11	9/10	0/11	10/10	1/11	8/10

Table shows number of animals with positive immunostaining/total number of animals and group at different time points. Control (C57BL/6) and DKO (apoE^-/-^/LDLR^-/-^) mice.

**Figure 1:**
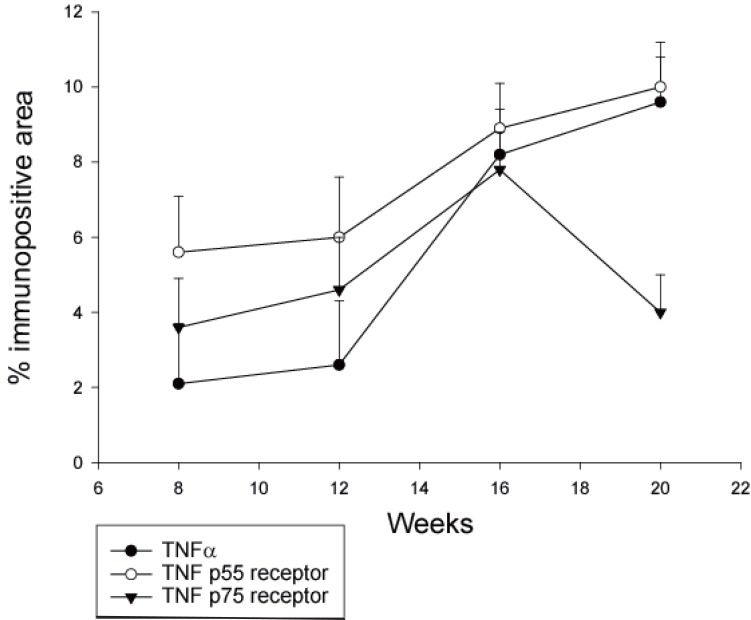
Percent of media covered by TNF-α and TNF receptor immunoreactivity in apoE^-/-^/LDLR^-/-^ mice. Immunohistochemical staining for TNF-α, TNF p55 and p75 receptors were performed as described in the *Methods* section. A mean medial staining for each animal was calculated based on analyses of 2 sections from the aortic arch, 2 from the descending thoracic aorta and 2 from the abdominal aorta. Each value represents the mean ± SD of 5 (8 and 12 weeks of age) or 10 (16 and 20 weeks of age) mice.

**Figure 2:**
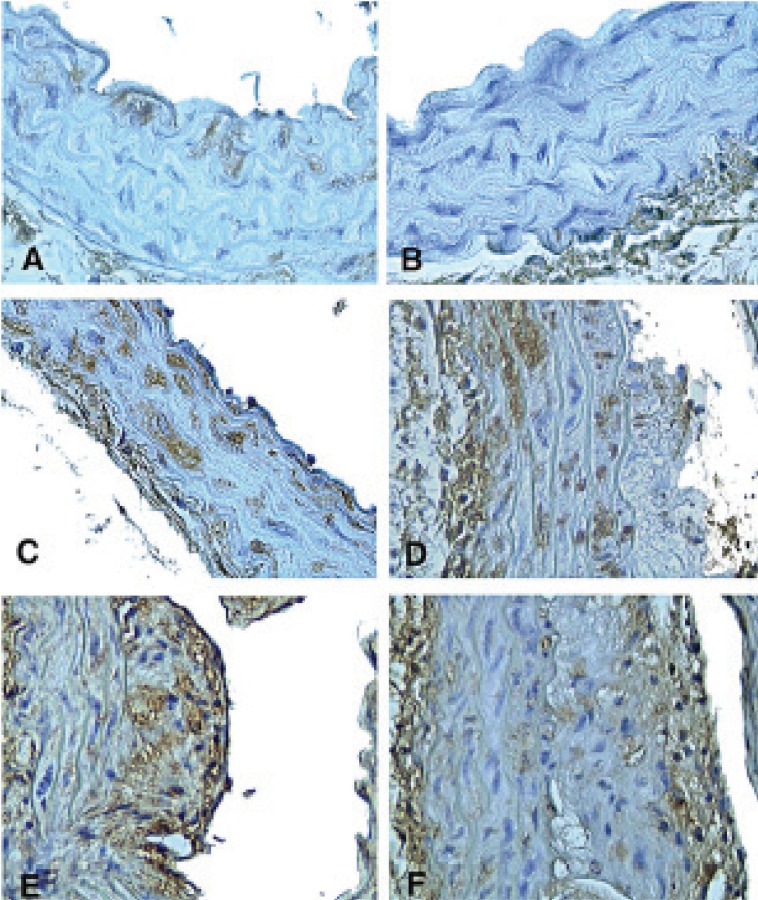
Expression of TNF-α and TNF receptor immunoreactivity in the aorta of apoE^-/-^/LDLR^-/-^ mice and C57BL/6 mice. Micrographs demonstrating (A) subendothelial immunostaining of TNF-α in a 12-week old apoE^-/-^/LDLR^-/-^ mouse, (B) absence of TNF-α expression in a 20-week old C57BL/6 mouse, (C) expression of TNF-α throughout the media and (D) in the media and in an atherosclerotic plaque in a 20-week old apoE^-/-^/LDLR^-/-^ mouse, (E) expression of TNF p55 receptor and (F) TNF p75 receptor immunoreactivity in an atherosclerotic plaque in a 20-week old apoE^-/-^/LDLR^-/-^ mouse. Original magnification x 400 in A-C and x 200 in D-F.

**Figure 3:**
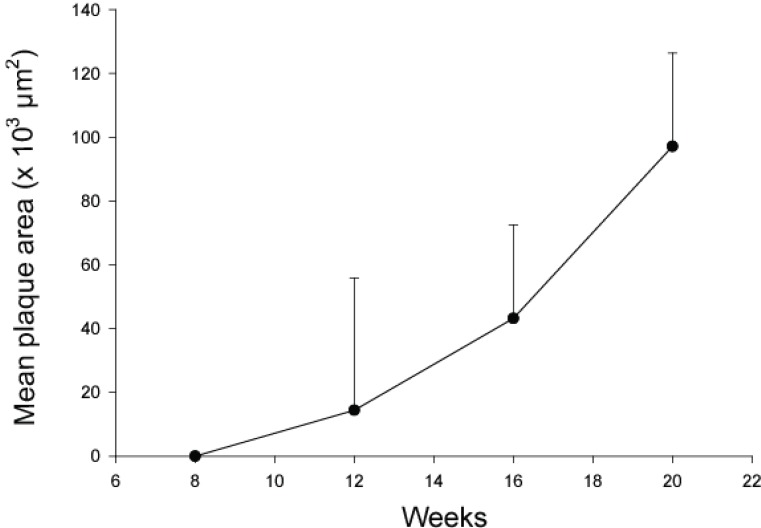
Plaque development in apoE^-/-^/LDLR^-/-^ mice. A mean plaque area for each animal was calculated based on analyses of 6 sections from the aortic arch, 6 from the descending thoracic aorta and 6 from the abdominal aorta. Each value represents the mean ± SD of 5 (8 and 12 weeks of age) or 10 (16 and 20 weeks of age) mice.

At 8 weeks of age TNF-α immunostaining covered 2.1 ± 1.6% of the aortic media in apoE^-/-^/LDL receptor^-/-^ mice. TNF p55 receptor immunoreactivity covered 5.6 ± 1.5% and TNF p75 receptor immunoreactivity 3.6 ± 1.3% of the media (Figure 1). Aortas of these 8-week old animals did not contain detectable atherosclerotic plaques. At 12 weeks of age, immunoreactivity for TNF-α and TNF receptors had increased only marginally. The expression of immunoreactivity was cell-associated (Figure 2). Atherosclerotic plaques were observed in 1 out of 5 mice. At 16 weeks TNF-α immunoreactivity was increased more than four-fold as compared with 8 weeks (*P*<0.001) and also the expression of TNF p55 and p75 receptors was significantly increased as compared with 8 week old animals (*P*<0.01 and *P*<0.05, respectively; Figure 1). Widespread atherosclerosis was present in most of the 16-week old animals. At 20 weeks, mean plaque size had more than doubled as compared to 16 weeks old mice (Figure 3). The expression of TNF-α and the TNF p55 receptor was also further increased, but not to the same extent as the plaque size (Figure 1). There were no significant differences in the expression of TNF-α or its receptors between animals that had developed detectable atherosclerosis and those that had not.

The expression of TNF-α and TNF receptor immunoreactivity was generally more abundant in plaques than in the media in the double knock-out mice (Table 3). In foam cell-rich lesions of fatty streak-type, TNF-α and TNF receptor were present throughout the entire lesion. In more advanced plaques, TNF-α and TNF receptor immunoreactivity was usually observed in the shoulder region and in the fibrous cap, but not close to the proximity of necrotic cores (Figure 2). In the media, increased expression of TNF-α was often present in SMC located immediately under an advanced plaque (Figure 2).

**Table 3:** Plaque size, TNF-α and TNFR expression in apoE^-/-^/LDLR^-/-^ mice at 16 and 20 weeks of age

	16 weeks	20 weeks

Plaque size	43 ± 11.5	97.1 ± 41.4
Media TNF-α	8.2 ± 1.0	9.6 ± 1.2
Media TNF p55 receptor	8.9 ± 1.0	10.0 ± 1.1
Media TNF p75 receptor	7.8 ± 1.1	4.0 ± 0.8
Plaque TNF-α	12.4 ± 2.6	12.3 ± 1.8
Plaque TNF p55 receptor	19.1 ± 4.7	22.6 ± 2.4
Plaque TNF p75 receptor	9.9 ± 3.0	14.8 ± 2.5

Values are presented as mean ± standard deviation of plaque size (× 10^3^ µm^2^) and immunostaining for TNF-α and TNF receptors (% of total media or plaque area) (n=10 per group).

The amounts of TNF-α mRNA transcripts assessed by Real Time PCR decreased with age in both apoE^-/-^/LDL receptor^-/-^ and control mice (Table 4). In 16-week old mice there was a tendency towards higher TNF-α mRNA levels in the double knock-out mice compared to controls (*P*=0.06). No significant differences in expression of TNF p55 and TNF p75 receptor mRNA levels were detected in the double knock-out mice compared to controls (Table 4). There was a high variability in the mRNA expression ratios within all groups. This variability may explain the inconsistency between the mRNA expression and the immunohistochemistry staining for TNF p75. A stronger TNF p75 staining was found in apoE^-/-^/LDL receptor^-/-^ mice than in controls (Table 2), whereas no such difference in the mRNA levels was detected (Table 4).

**Table 4:** TNF-α and TNFR mRNA expression in apoE^-/-^/LDLR^-/-^ mice

	TNF-α		TNF p55 receptor		TNF p75 receptor	
Age (weeks)	Control	DKO	Control	DKO	Control	DKO

16	852 ± 987	3005 ± 1998	468 ± 326	392 ± 270	1495 ± 1245	412 ± 433
20	106 ± 174	52 ± 74	462 ± 355	1511 ± 2856	1174 ± 884	232 ± 229

Values are given as the ratio of TNF-α or TNF receptor mRNA expression, respectively, and 18S rRNA expression. Control; C57BL/6 mice, DKO; apoE^-/-^/LDLR^-/-^ mice (n=10-11 per group).

## DISCUSSION

The present study demonstrates that vascular expression of TNF-α and TNF p55 and p75 receptors precedes development of atherosclerotic plaques in apoE^-/-^/LDL receptor^-/-^ mice. The expression is mainly located in vascular media. The medial TNF-α and TNF receptor expression in apoE^-/-^/LDL receptor^-/-^ mice increased with age and in parallel with progression of lesions with the exception of the TNF p75 receptor that showed decreased staining at 20 weeks. Virtually no TNF-α or TNF receptor immunoreactivity was detected in C57BL/6 control mice. This may be an explanation for why depletion of TNF receptors in this non-atherosclerosis prone mouse model doesn’t fit other observations that TNF-α would be a pro-atherogenic cytokine (21).

A medial expression of TNF-α in non-atherosclerotic arteries of hypercholesterolemic animals such as the apoE^-/-^/LDL receptor^-/-^ mouse is well in line with previous studies showing expression of TNF-α mRNA and protein in medial SMC of 6 month old WHHL rabbits (25). Using an animal model in which human LDL is injected intravenously into rats we have previously shown that accumulation of LDL in rat aorta is associated with enhanced TNF-α mRNA and protein expression within 12 hours (24). At this time point arterial LDL display characteristics specific for oxidative modification (26). However, cell culture experiments suggest that it is native rather than oxidized LDL that is responsible for induction of SMC TNF-α production (24).

Only a sub-population of medial SMC appears to express TNF-α and TNF receptors in response to hypercholesterolemia. Studies in balloon-injured rabbits have provided evidence suggesting that medial TNF-α is primarily produced by proliferating SMC (17). It has been proposed that SMC engaged in arterial repair processes and in the formation of atherosclerotic plaques are recruited from a distinct sub-population of medial SMC with increased proliferative capacity (27). Such cells have been isolated from the neointima of balloon-injured rats as well as from normal media (28, 29). It is possible that the TNF-α expressing medial SMC observed in the present study represent such a sub-population of cells. Indeed, we have previously demonstrated that SMC isolated from rat neointima have increased levels of TNF-α and TNF receptors in culture (30). TNF-α does not appear to be involved in the increased rate of proliferation in these cells; instead an endogenous activation of TNF receptors induced cell death by apoptosis limiting the proliferative response.

Previous studies of vascular inflammatory response to hypercholesterolemia have mainly focused on early up-regulation of adhesion molecules in the endothelium. Our present findings demonstrate presence of an early inflammatory reaction also in the media. The functional role of this inflammatory response remains to be elucidated, but it is likely to participate in activation of endothelial adhesion molecule expression. One possible scenario is that medial SMC respond to extensive accumulation of native, aggregated or oxidized LDL particles with stimulated TNF secretion resulting in signals to the endothelium to recruit monocytes for modified lipoprotein removal.

In established lesions accumulation of B cells occurs primarily at sites where the surrounding cells express TNF-α and VCAM-1, suggesting that TNF-α-induced expression of adhesion molecules may be of importance for recruitment of mononuclear cells also within plaques (31).

An important question is whether TNF-α in itself plays a critical role in the development of atherosclerosis. This issue is made even more relevant by the recent development of effective TNF-α-inhibitors for use in other chronic inflammatory diseases. TNF-α-inhibition could influence concomitant cardiovascular disease or even be used directly for treatment of atherosclerosis (5). A substantial body of experimental evidence supports the notion that TNF-α has pro-atherogenic effects. It induces endothelial activation (14, 32), stimulates the release of pro-inflammatory cytokines (33) and matrix metalloproteinases (34), activates SMC migration and proliferation (18, 23), inhibits collagen synthesis (35), initiates apoptosis (16) and inhibits fibrinolysis by stimulating the release of plasminogen activator inhibitor (PAI)-1 (36). Animal studies have provided more conflicting results. TNF-α inhibition reduces transplant arteriosclerosis in rabbits (19) and the size of atherosclerotic lesion in apo E deficient mice (20), whereas mice lacking either of the TNF p55 and p75 receptors do not show reduced lesion formation (21). The present observation that vascular expression of TNF-α and TNF receptors precedes plaque formation in this mouse model of atherosclerosis provides some indirect support for a pro-atherogenic role of TNF-α. The most pronounced increase in medial TNF-α expression occurred at 16 weeks of age, at the same time as the highest TNF-α mRNA levels was detected and as a more wide-spread atherosclerosis began to develop. This may suggest that medial TNF-α expression is actively involved in the disease process, but may also reflect pro-inflammatory effects of plaques on the underlying media.

In summary, this study shows that medial TNF-α and TNF receptor expression precedes lesion formation in the atherosclerosis-susceptible apoE^-/-^/LDL receptor^-/-^ mouse, whereas the aortas of control C57BL/6 mice were essentially negative for TNF-α and TNF receptor immunoreactivity. This suggests that lipid-induced activation of TNF-α expression may play a role in endothelial activation and other inflammatory processes during early stages of atherosclerosis.
